# Reconfigurable Architecture and Dataflow for Memory Traffic Minimization of CNNs Computation

**DOI:** 10.3390/mi12111365

**Published:** 2021-11-05

**Authors:** Wei-Kai Cheng, Xiang-Yi Liu, Hsin-Tzu Wu, Hsin-Yi Pai, Po-Yao Chung

**Affiliations:** Department of Information and Computer Engineering, Chung Yuan Christian University, Taoyuan City 32023, Taiwan; g10777002@cycu.edu.tw (X.-Y.L.); g10977035@cycu.edu.tw (H.-T.W.); g11077001@cycu.edu.tw (H.-Y.P.); g10977024@cycu.edu.tw (P.-Y.C.)

**Keywords:** CNN, DRAM, PE array, dataflow, data migration, data reuse

## Abstract

Computation of convolutional neural network (CNN) requires a significant amount of memory access, which leads to lots of energy consumption. As the increase of neural network scale, this phenomenon is further obvious, the energy consumption of memory access and data migration between on-chip buffer and off-chip DRAM is even much more than the computation energy on processing element array (PE array). In order to reduce the energy consumption of memory access, a better dataflow to maximize data reuse and minimize data migration between on-chip buffer and external DRAM is important. Especially, the dimension of input feature map (ifmap) and filter weight are much different for each layer of the neural network. Hardware resources may not be effectively utilized if the array architecture and dataflow cannot be reconfigured layer by layer according to their ifmap dimension and filter dimension, and result in a large quantity of data migration on certain layers. However, a thorough exploration of all possible configurations is time consuming and meaningless. In this paper, we propose a quick and efficient methodology to adapt the configuration of PE array architecture, buffer assignment, dataflow and reuse methodology layer by layer with the given CNN architecture and hardware resource. In addition, we make an exploration on the different combinations of configuration issues to investigate their effectiveness and can be used as a guide to speed up the thorough exploration process.

## 1. Introduction

With the rapid development of artificial intelligence, CNN is often used in various applications of artificial intelligence, such as machine learning, computer vision, computational neuroscience, etc. Although the function of the convolutional neural network is more and more powerful, it is accompanied by a large number of convolution operations, and a large amount of memory access and data migration is also required. As the increasing number of layers of neural network, the memory access issue becomes more and more important.

Especially in the edge computation, a large amount of memory access is often a bottleneck that affects the performance and energy consumption of hardware accelerators. Different from the memory access method of CPU or GPU architecture, processing the transfer of data between neural operators in a dataflow method is a common way to implement CNN hardware accelerators. In this spatial type of CNN hardware accelerator architecture, ways to improve memory access efficiency and reduce energy consumption include (1) increase the reusability of data in the neural computing cell network; (2) reduce the cost and energy consumption of data migration; (3) reduce access to the external memory of the chip. Therefore, dataflow planning, processing elements (PE) and buffer configuration are often used to reduce data migration and energy consumption of memory in the hardware implementation.

**Figure 1:**
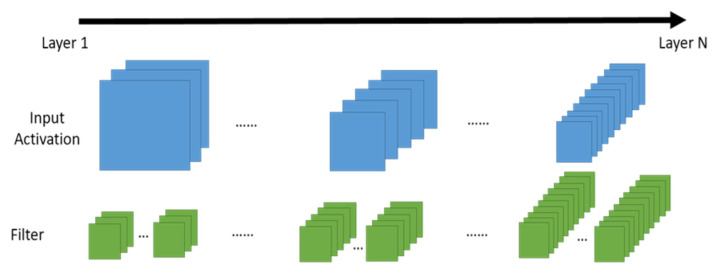
Dimension shape of a CNN.

There have been a lot of studies related to the PE architecture design, interconnect and dataflow configuration of CNNs. Research [1] summarized well known dataflow include input stationary, weight stationary, and output stationary. Research [2,3] proposed dataflow enhancement techniques. Research [4,5,6] especially targeted their dataflow optimization on the systolic array architecture. Research [7,8] addressed the hardware reconfiguration problem. However, all the previous research uses a fixed PE array configuration through the entire neural network computation, and does not take into account the configuration of PE array structure, buffer assignment, and dataflow approach simultaneously. Furthermore, the configuration and aspect ratio of ifmap dimension and filter dimension are much different among all the CNN layers, but this issue is seldom considered in the previous research. As the modern CNN architecture shown in Figure 1, the first layer has the largest ifmap dimension and the smallest filter dimension; while in the latter layers the ifmap dimension decreased and the filter dimension increased.

In this paper, we propose a quick and efficient methodology to adapt the configuration of PE array architecture, buffer assignment, dataflow and reuse methodology layer by layer with the given CNN architecture and hardware resource. In addition, we make an exploration on the different combination of configuration issues to investigate their effectiveness, and can be used as a guide to speed up the thorough exploration process. To the best of our knowledge and survey, we are the first ones to address the problem of adapting PE array architecture, buffer assignment, and dataflow approach simultaneously for CNNs.

The rest of this paper is organized as follows. In Section 2, we introduce the background and motivation of our reconfiguration proposal for PE array architecture, buffer assignment and dataflow approach. Section 3 describes the platform and methodology for our configurable PE array architecture, buffer management, and dataflow with compound data reuse. Section 4 shows our evaluation methodology and experiment results. In Section 5, we analyze and discuss the exploration results on different architecture configurations. Finally, we draw the conclusions and future works in Section 6.

## 2. Background and Motivation

### 2.1. Preliminary

The entire CNN dataflow starts from the input activations of the first layer to the output activations of the last layer, we can regard it as a data stream. The most basic operation in CNN is multiply-and-accumulate (MAC), how to make MAC in the network can be calculated in parallel becomes an important issue in the design of CNN hardware accelerator, and it is also dedicated to both temporal architecture and spatial architecture.

In temporal architectures such as CPU or GPU, common parallelization technologies include vector (SIMD) or parallel sequence (SIMT). A single core controller uniformly controls all computing units in the CNN network. Data access and transmission are used with the hierarchical memory architecture of traditional computers, various computing units cannot directly communicate and transmit information. In addition to parallelization technology, because CNN requires a large number of matrix multiplication calculations, how to map these matrix calculations to convolution or fully connected network architecture, and use Fast Fourier Transform (FFT) [9] or other conversion methods [10,11] to reduce the number of matrix calculations, and select the appropriate conversion algorithm according to the shape and size of the matrix [12,13], which are the main techniques of temporal architecture to improve the performance of CNN operations.

In contrast, spatial architecture increases parallelism by means of dataflow. The computing units in the CNN network form data links. Data is directly transmitted between the computing units in accordance with the designed flow direction. At the same time, each computing unit has independent logic control circuit and local memory. This spatial architecture oriented by considering dataflow is mainly implemented in ASIC, FPGA-based, and applied to the design of CNN hardware accelerators for edge devices. Therefore, how to increase the data reusability of the local memory of each computing unit to reduce the energy consumption of data migration and correspondence is the focus of the energy-saving technology of spatial architecture in CNN network computing.

In the spatial architecture, dataflow strategy is one of the most important issues to reduce external memory traffic. The most common dataflow strategy includes input stationary, weight stationary, output stationary, and row stationary. Input stationary, weight stationary, and output stationary are single data resident. With dataflow and memory mapping, input stationary, weight stationary, and output stationary respectively enable ifmap, weight, and partial sum to reside in the high-speed register of each processing element. In contrast, row stationary is the permanent residence of composite data. After a three-step process, it comprehensively maximizes the data reusability of input weight, pixel, and partial sum.

In addition to dataflow strategy, how to increase the reusability of data to reduce data migration is another important consideration in the design of CNN hardware accelerators. CNN operations can have three forms of data reuse: convolutional reuse, feature map reuse, and filter reuse. In convolutional reuse, the same activations and filter weights are in the same channel, and are reused in different combinations to produce different calculation results. In feature map reuse, multiple groups of filter weights are applied to the same feature map, so a feature map activations will be repeatedly calculated by different filters. In filter reuse, a set of filter weights are applied to different feature maps, so a set of filter weights will be repeatedly calculated with different feature maps.

### 2.2. Related Works

The core point of weight stationary technology is to increase the reusability of the data in the local memory when reading the filter weight, and reduce the number of reads and writes because of the need for off-chip DRAM. Therefore, through the arrangement and optimization of the dataflow, the filter weight is permanently resident in the local memory. The ifmap activation is transmitted to each arithmetic unit by broadcasting, and the partial sum relies on the dataflow between the arithmetic units for the accumulation operation. The CNN related documents that apply this weight stationary dataflow technology include neuFlow et al. [14,15,16,17,18,19].

Compared with weight stationary, the core point of output stationary technology is to increase the resident of partial sum belonging to the same output activation in the local memory during the accumulation process. In order to achieve this goal, basically, when the dataflow is arranged and optimized, the input activation is passed between the computing units along with the dataflow, and the filter weight is transmitted to each computing unit by broadcasting. With the changes of different channel processing methods, related CNN documents that apply this output stationary data stream technology include ShiDianNao et al. [20,21].

Unlike weight stationary and output stationary that only consider the filter weight or partial sum of the data resident, the core point of row stationary technology is to increase the overall resident of all data types in the local memory to achieve the maximum memory energy saving effect. Therefore, row stationary is more complicated than the previous data streaming technologies in hardware implementation. The CNN related literature that applies this row stationary data stream technology includes Eyeiss et al. [22,23,24].

Some other research further enhanced the techniques on dataflow and data reuse, these methods include memory-centric design flow to optimize on-chip memory size and data reuse [2], fused-layer dataflow to fuse the processing of adjacent CNN layers by a multilayer sliding window [3,25], block convolution methodology to eliminate data dependencies between adjacent tiles [26], and a hierarchical exploration strategy on the dataflow configurations [27]. These studies show that the effects of the memory bottleneck can be reduced by a flexible memory hierarchy that supports the complex data access patterns in CNN workload, and the efficiency of the on-chip memories is maximized by their scheduler that uses tiling to optimize data locality.

As the dataflow of the systolic array is efficient for CNN computation, some dataflow optimization techniques specially targeted on the systolic array architecture [4,5,6]. In this research, adaptive layer partitioning and scheduling schemes were proposed to minimize off-chip memory access, they can adaptively switch among different data reuse schemes and the corresponding tiling factor settings to dynamically match different convolutional layers.

Except for configuration techniques on dataflow to increase data reuse, some other research addressed the problem of hardware reconfiguration and the corresponding compiler optimization techniques [6,7,28,29,30]. They proposed to build a set of modular and configurable building blocks, reconfigure the microarchitecture parameters of the accelerator dynamically, and introduced a set of data-centric directives to concisely specify the CNN dataflow space in a compiler friendly form.

Finally, since FPGA is a promising platform for running CNN algorithm, research [31] proposed a scheduling algorithm and data reuse system to optimize on-chip memory usage for on-board FPGA. Because of limited FPGA storage capability and memory bandwidth, different configurations allow either to minimize on-chip memory usage or maximize memory bandwidth.

In summary, there have been many studies on the reconfiguration and optimization methodologies for dataflow and hardware architecture of CNN, respectively. However, to the best of our knowledge and survey, we are the first ones to propose a thorough exploration of the reconfiguration of systolic architecture, internal buffer, dataflow and reuse techniques. In the next subsection, we present a data analysis to show the motivation why we address this problem.

### 2.3. Motivation

**Figure 2:**
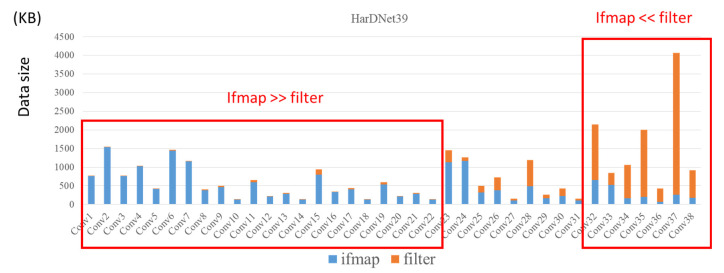
Data size distribution of HarDNet39.

As shown in Figure 1, the first layer has the largest ifmap dimension and the smallest filter dimension, and in latter layers their ifmap dimension decreased and filter dimension increased. Therefore, aspect ratio of ifmap dimension and filter dimension are much different among CNN layers. Figure 2 shows the data size distribution of HarDNet39 [32], we see that data size of ifmap is much larger than data size of filter in former layers, and vice versa in latter layers.

**Figure 3:**
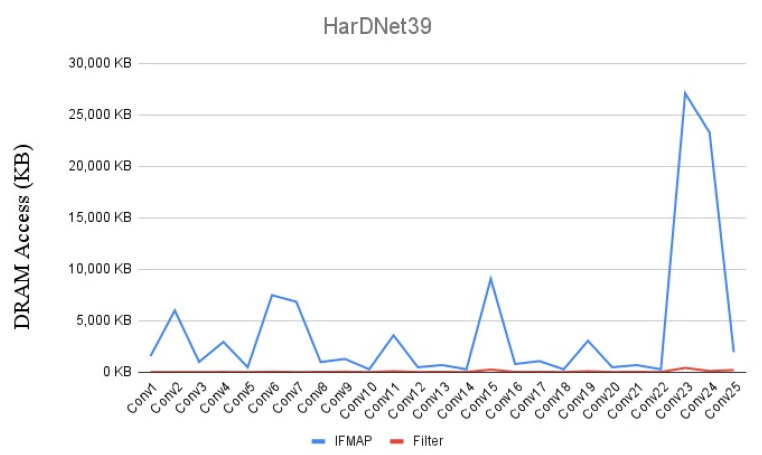
DRAM access distribution of HarDNet39.

As described above, because the data size of ifmap dimension and filter dimension are much different among CNN layers, a single dataflow and data reuse method cannot fit well for both ifmap dimension and filter dimension in all layers. Figure 3 shows the DRAM access of the first twenty-five layers of HarDNet39 from the SCALE-Sim [33] simulator. The configuration parameters of this evaluation is 16 × 16 PE array, 50 kB buffer for both ifmap and filter, and the applied dataflow is output stationary with convolutional reuse. As the dimension of ifmap is much larger than the dimension of filter weights in the first twenty-five layers of HarDNet39, and the reuse methodology is convolutional reuse which does not benefit either ifmap reuse or filter reuse, we see that only a little data migration of filter weight in these layers, but there is a large quantity of data migration of ifmap. Therefore, since data dimension of ifmap is much larger than filter in these layers, increase data reuse of filter weight is not a good choice to reduce total DRAM access. In contrast, if we use ifmap reuse methodology for these layers, although it will increase a little data migration of filter weight, but we can reduce further much more data migration of ifmap, and hence reduce the total DRAM access.

In summary, a fixed configuration of architecture and dataflow cannot fit well for all CNN layers. However, a thorough exploration on all possible configurations is time consuming and meaningless. Therefore, we propose a quick and efficient methodology to adapt the configuration of PE array architecture, buffer assignment, dataflow and reuse methodology layer by layer with the given CNN architecture and hardware resource. In addition, we make an exploration on the different combination of configuration issues to investigate their effectiveness, and can be used as a guide to speed up the thorough exploration process.

## 3. Methodology

**Figure 4:**
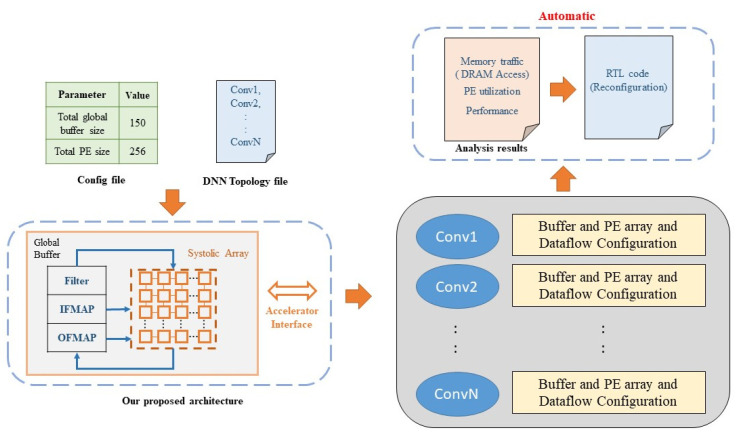
The proposed exploration platform.

In this section, different from SCALE-Sim [33] that cannot configure architecture and dataflow layer by layer, we introduce our exploration platform and configuration methodology for layer by layer configuration. As shown in Figure 4, input to the platform includes a configuration file and a topology file, the configuration file specifies the total number of PE and total buffer size of ifmap and filter, and the topology file specifies the topology of ifmap and filter of each CNN layer. After configuration, our platform report the configuration result of each CNN layer, and based on the trace simulation we can report memory access and PE utilization of each layer and the summation of total layers.

There are three types of configuration in this platform, PE array configuration, buffer assignment of ifmap and filter, configuration of dataflow and data reuse methodology. The three configuration items can be selected in any combination, if no configuration item is selected then all CNN layers will have the same fixed architecture and dataflow configuration. In the fixed configuration style, the input configuration file can specify PE array dimension, individual buffer size of ifmap and filter. However, the dataflow in the fixed configuration style is fixed to output stationary with convolution reuse since it needs no extra output buffer to store partial sum, and as the experiment results shown in SCALE-Sim [33] that it also has minimal off-chip data migration during convolutional computation.

In contrast, when the layer by layer item of dataflow configuration is selected, although we also fix to the output stationary dataflow, but we can configure ifmap reuse or filter reuse methodology layer by layer. Another reason why we didn’t make input station and weight stationary dataflow as the candidate of layer by layer configuration is because that change of dataflow between, output stationary, input stationary and weight stationary will increase the design complexity and control overhead of PE architecture, but with almost no benefit on reducing external memory access. While for our methodology that fix to output stationary dataflow but changes the data reuse technique as necessary will not increase the design complexity of PE architecture, and also will have the least control overhead.

Finally, when the layer by layer configuration item is selected for total PE array or total ifmap/filter buffer, our platform also configure the PE array dimension and ifmap/filter buffer assignment layer by layer. Detailed methodology for the three types of configuration in our platform is described in the following subsections.

### 3.1. PE Array Configuration

Principle of our PE array configuration method is defined as below: Under the total PE number k (k is index of 2), we determine the PE array configuration of each layer based on their aspect ratio of ifmap dimension and filter dimension, such that for each layer, m * n = k, m, n, k ∈ 2^i^, i ≥ 1, i ∈ Z, where m and n denote the row number and column number of PE array configuration.

**Figure 5:**
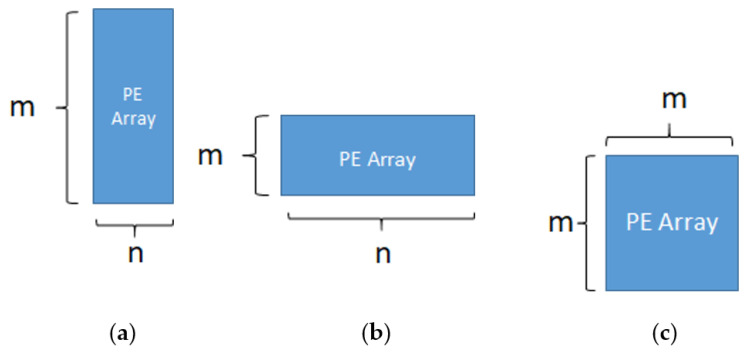
Concept of PE array configuration (**a**) ifmap > filter (**b**) ifmap < filter (**c**) ifmap ≒ filter.

Figure 5 illustrates the concept of our methodology for PE array configuration, ifmap is passed in from left to right in the horizontal direction, and filter weight is passed in from top to bottom in the vertical direction to perform MAC (multiply and accumulate) operations in order. For the layer with ifmap greater than filter * t_1_, we set array configuration m > n as shown in Figure 5a. In contrast, for the layer with filter greater than ifmap * t_2_, we set array configuration m < n as shown in Figure 5b. Finally, for others that do not belong to the above two, we set array configuration m = n as shown in Figure 5c. The parameter value t_1_ and t_2_ can be different for different PE number k.

**Figure 6:**
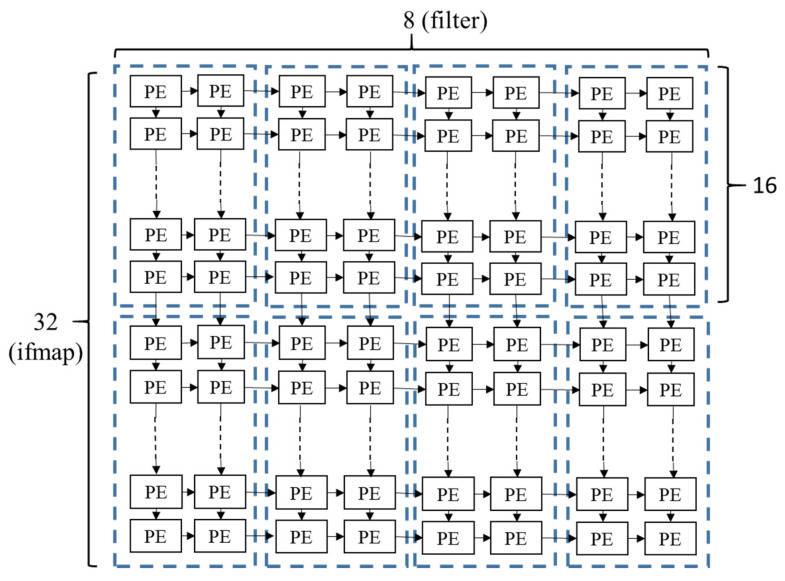
Cluster-based PE array configuration.

To realize the proposed concept of PE array configuration in Figure 5, we propose a cluster-based array architecture. For the example of total 256 PE number, the possible configuration can be 128 × 2, 64 × 4, 32 × 8, 16 × 16, 8 × 32, 4 × 64, and 2 × 128. After layer by layer configuration of a CNN, if the selected configurations are 128 × 2, 64 × 4, 32 × 8, and 16 × 16, then we will make a PE cluster by 16 × 2 sub-array as shown in Figure 6. By different cluster connection, we can support all the four selected PE array configurations in different layers as their need. Figure 6 is an example of 32 × 8 PE array implement with 16 × 2 cluster-based architecture. We can adjust the cluster connection to easily implement the 128 × 2, 64 × 4, and 16 × 16 PE array architecture with the same 16 × 2 PE cluster, and the overhead of control logic is quite small in comparison with the PE architecture and circuit design.

In summary, our configuration method allows each layer of CNN to have its own PE array configuration based on the dimension of ifmap and filter. As it is not limited to a fixed configuration all through the entire network, thereby can improve the overall PE utilization and reduce the external memory traffic.

### 3.2. Buffer Configuration

**Figure 7:**
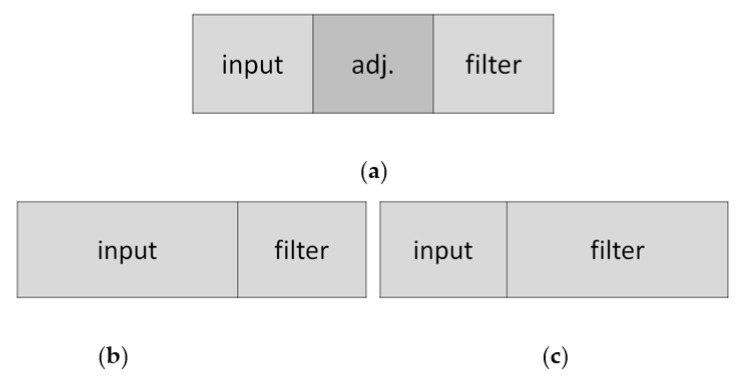
Concept of adjustable buffer configuration (**a**) adjustable buffer (**b**) buffer configuration for ifmap > filter (**c**) buffer configuration for ifmap < filter.

If we can make use of buffer reconfiguration in the accelerator design to maximize data reuse, we can avoid rereading data from external DRAM and speedup the overall CNN computation. Therefore, under the given of total global buffer size, we then cut the input buffer and filter buffer according to the dimension of the PE array configuration. In our method, the buffer configuration is adjustable layer by layer as shown in Figure 7a. In addition, because the output feature map calculated for each layer will be written back to the external DRAM through the output buffer, our output buffer will be set to a fixed size, and the output buffer will not be dynamically configured. 

For each layer, the principle of our buffer configuration is described as below. In the case that the data size of ifmap is much larger than that of filter, the input buffer size will be configured to be larger than the filter buffer size as shown in Figure 7b. On the other hand, the input buffer size will be configured to be smaller than the filter buffer size as shown in Figure 7c. Finally, when the difference of data size is small, the input buffer size will be configured to be the same with the filter buffer size.

For the illustration example, if the given total PE number is 256, the possible PE array configurations can be 128 × 2, 64 × 4, 32 × 8, 16 × 16, 8 × 32, 4 × 64, and 2 × 128. Under 100 KB of total buffer size for ifmap and filter, our configuration of (ifmap buffer, filter buffer) can be (87.5 KB, 12.5 KB), (75 KB, 25 KB), (62.5 KB, 37.5 KB), (50 KB, 50 KB), (37.5 KB, 62.5 KB), (25 KB, 75 KB), and (12.5 KB, 87.5 KB), total seven configurations to match with the PE array configurations. In this way, not all layers are assigned to a fixed equal buffer configuration, which can avoid unnecessary external data access for layers that have much different ifmap dimension and filter dimension.

### 3.3. Dataflow and Data Reuse Configuration

**Figure 8:**
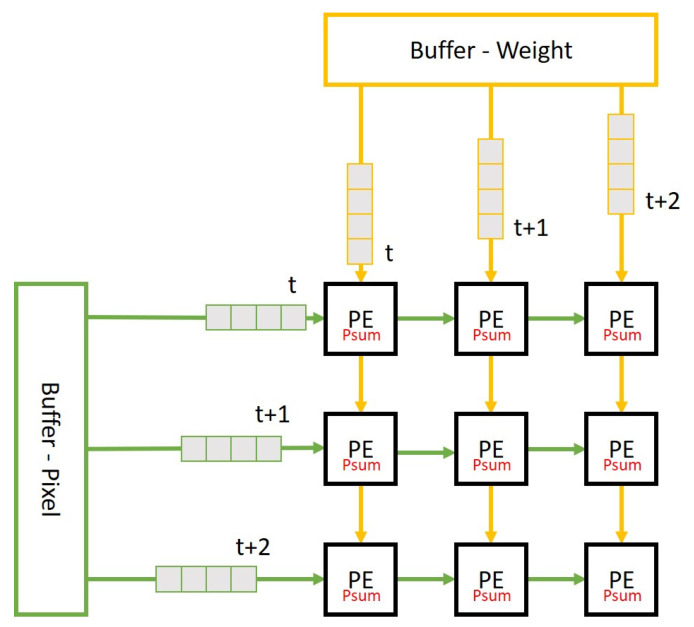
The output stationary dataflow.

Output stationary technique has the minimal data migration of partial sum, and hence has less total memory access in comparison with input stationary and weight stationary techniques. Therefore, output stationary is selected as the dataflow strategy for most of the CNN networks, Figure 8 shows the scheme of output stationary. In terms of data reuse technique, the output stationary dataflow can further be divided into convolutional reuse, ifmap reuse and filter reuse, and almost all previous works on output stationary dataflow use convolutional reuse technique only all through the entire CNN since it is a compromise between ifmap reuse and filter reuse.

However, for layers with an extreme large ifmap aspect ratio, the use of convolutional reuse dataflow will lead to a large quantity of ifmap data migration between external memory and internal buffer, and vice versa for layers with an extreme large filter aspect ratio. Therefore, in addition to convolutional reuse, we also integrate the other two reuse techniques in our output stationary dataflow. According to the PE array configuration of each layer, when the configuration is ifmap row >> filter column, we will use ifmap reuse technique, let ifmap fixing and replacing filters to reduce memory traffic of ifmap, and vice versa to use filter reuse technique when the PE array configuration is ifmap row << filter column.

**Figure 9:**
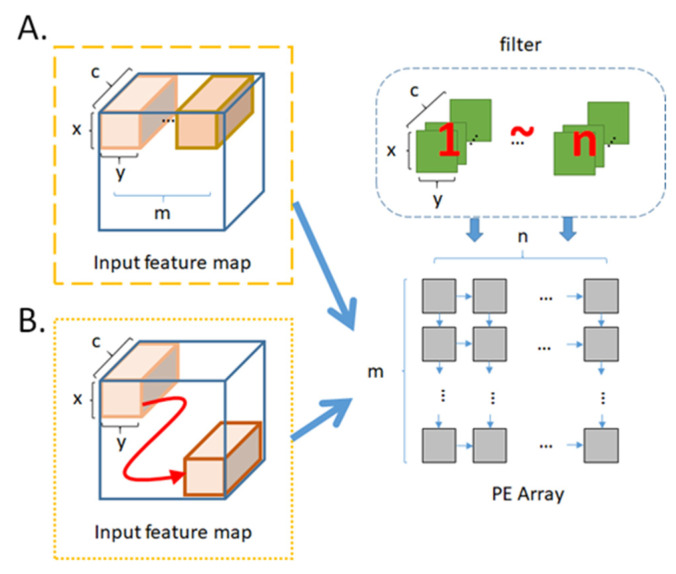
Procedure of filter reuse (**A**) the first iteration (**B**) the successive iterations.

Figure 9 illustrates the filter reuse technique. In the case that the size of PE array is m × n and the number of filter is r, let x, y, and c to be the length, width, and channel of the filter respectively. We read m sets of ifmap in order in the horizontal direction, and read n sets of filter in order in the vertical direction, such as in Figure 9A. After each round of convolution operation is completed, the n sets of filter are not replaced but replace the next batch of m sets of ifmap. This replacing procedure continues until the entire ifmap of this layer complete convolution operation, as shown in Figure 9B. Then we read the next n sets of filter, and repeat the steps in Figure 9 until all r sets of filter complete the convolution operation. For layers with large filter aspect ratio, we will adopt the filter reuse technique to replace convolutional reuse technique.

**Figure 10:**
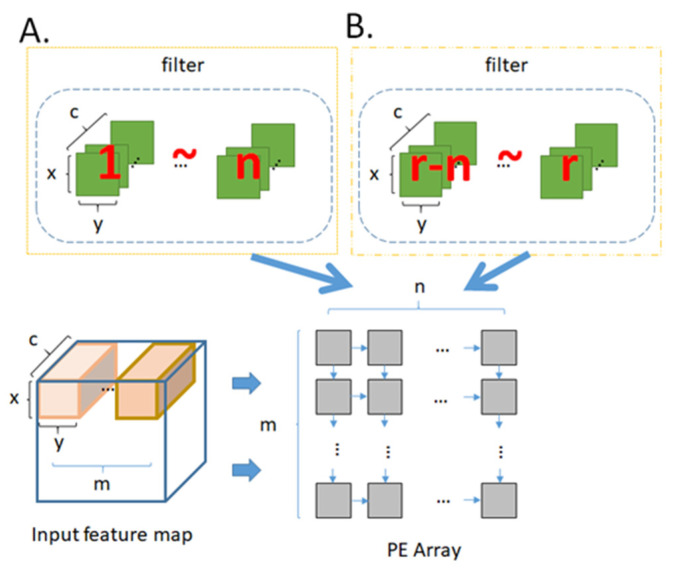
Procedure of ifmap reuse (**A**) the first iteration (**B**) the successive iterations.

In contrast to filter reuse technique, Figure 10 illustrates the ifmap reuse technique. We read m sets of ifmap in order in the horizontal direction, and read n sets of filter in order in the vertical direction, such as in Figure 10A. After each round of convolution operation is completed, the m sets of ifmap are not replaced but replace the next batch of n sets of filter. This filter replacing procedure continues until the all r sets of filter of this layer complete the convolution operation, as shown in Figure 10B. Then we read the next m sets of ifmap, and repeat the steps in Figure 10 until the entire ifmap complete the convolution operation. For layers with large ifmap aspect ratio, we will adopt the ifmap reuse technique to replace convolutional reuse technique.

## 4. Experiment Results

We modify SCALE-Sim [33] to evaluate our methodology on HarDNet39 [32] and DenseNet121 [34]. Table 1 shows the four target architecture configurations of the fixed dataflow and our proposed reconfigurable methods. In the fixed dataflow, all configurations are fixed in all layers, size of PE array are 16 × 16 and 32 × 32, respectively; input buffer and filter buffer are equally partitioned, total buffer size are 128 KB and 256 KB, respectively; dataflow is fixed to output stationary with convolutional reuse. While for our methodologies, architecture and dataflow are reconfigurable layer by layer based on the given total PE number and total buffer size of input and filter, dataflow is also fixed to output stationary but the reuse technique is configured based on aspect ratio of input dimension and filter dimension in each layer.

Table 2 shows the definition of configuration items and all possible exploration combinations in our platform. Since there are three configuration items: PE array, buffer size, dataflow and data reuse technique, totally there are eight combinations in our exploration platform. In this section, we will evaluate our methodology on HarDNet39 and DenseNet121 target to architectures list in Table 1. In the next section, we analyze and discuss these exploration results in terms of external memory access to show the effect of our configuration techniques.

**Figure 11:**
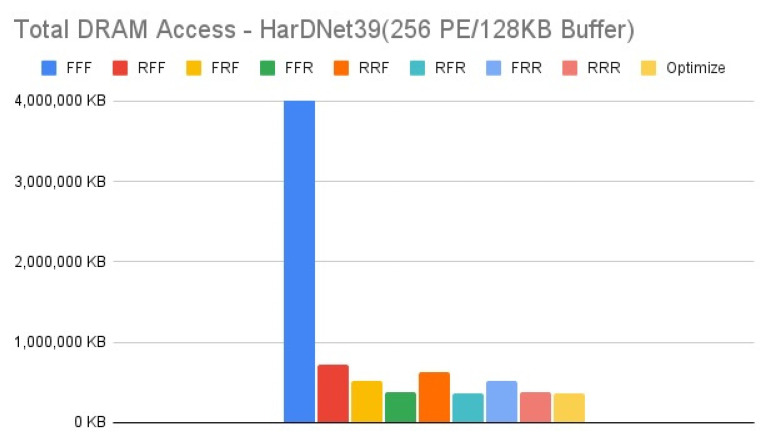
Total DRAM access—HarDNet39 (256 PE/128 KB Buffer).

**Figure 12:**
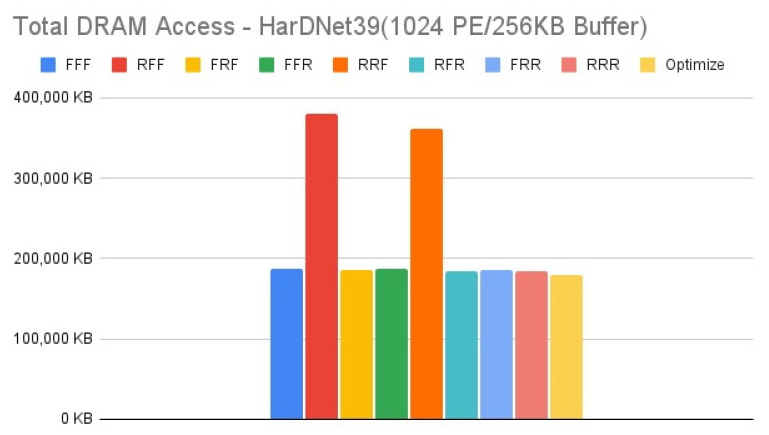
Total DRAM access—HarDNet39 (1024 PE/256 KB Buffer).

**Figure 13:**
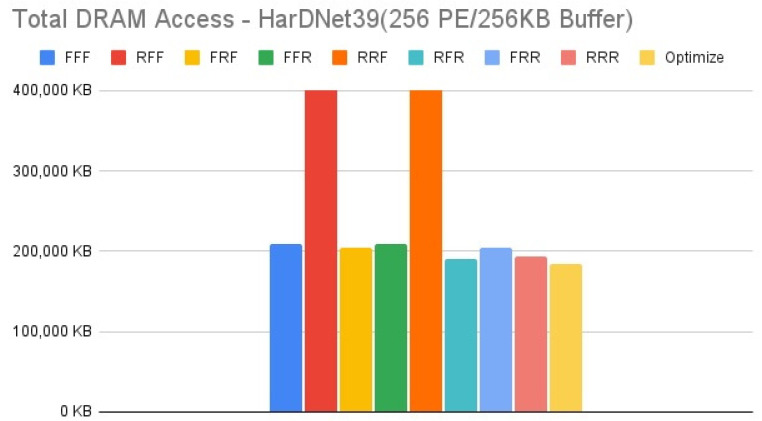
Total DRAM access—HarDNet39 (256 PE/256 KB Buffer).

**Figure 14:**
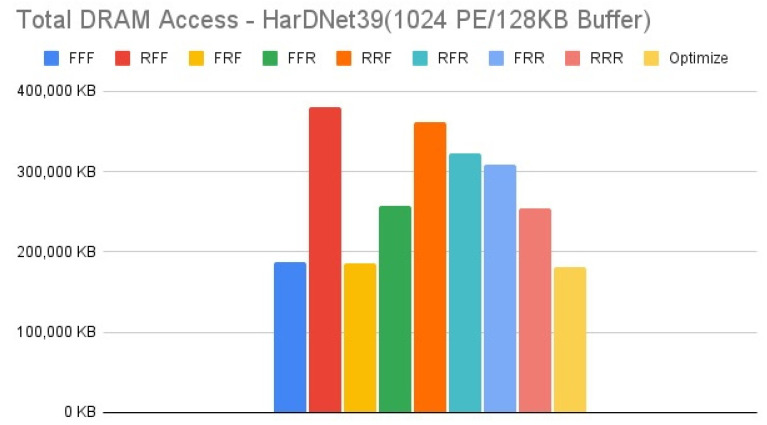
Total DRAM access—HarDNet39 (1024 PE/128 KB Buffer).

Figure 11, Figure 12, Figure 13 and Figure 14 show the exploration results of different configurations in terms of external memory access for HarDNet39 on the four target architectures. The “Optimize” item represents the result of adopting the best one of the eight configurations in each layer to get the total memory access, and hence has the best result in comparison with the eight configurations in our exploration platform. For the first target architecture, Figure 11 shows that the “FFF” configuration has the worst result. The second target architecture and the third target architecture have the similar configuration results, Figure 12 and Figure 13 show that the “RFF” and “RRF” configurations have even worse results than the “FFF” configuration. The fourth target architecture is an extreme case, Figure 14 shows that it has much different configuration results in comparison with the previous two target architectures. Detailed analysis and discussion will be given in the discussion section.

**Figure 15:**
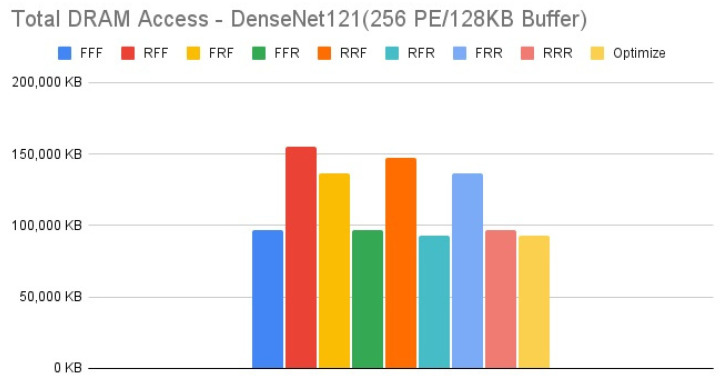
Total DRAM access—DenseNet121 (256 PE/128 KB Buffer).

**Figure 16:**
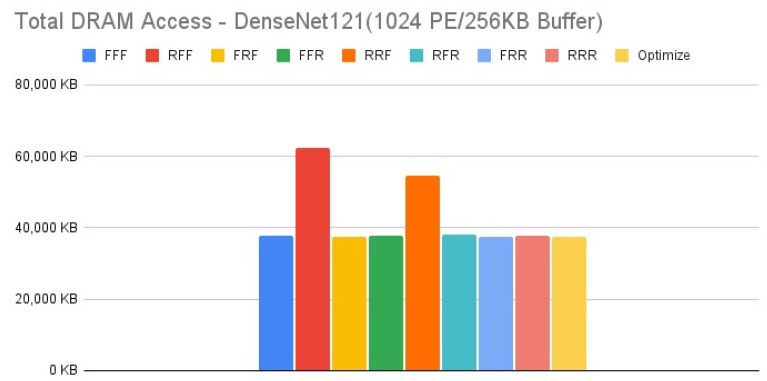
Total DRAM access—DenseNet121 (1024 PE/256 KB Buffer).

**Figure 17:**
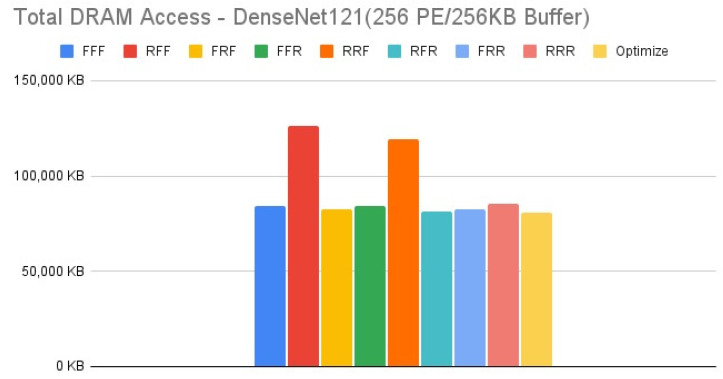
Total DRAM access—DenseNet121 (256 PE/256 KB Buffer).

**Figure 18:**
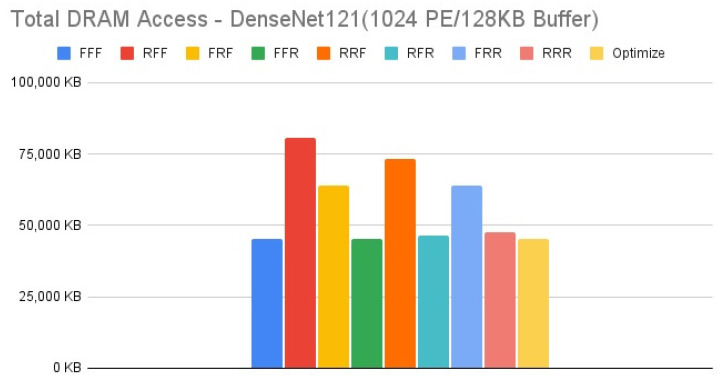
Total DRAM access—DenseNet121 (1024 PE/128 KB Buffer).

Figure 15, Figure 16, Figure 17 and Figure 18 show the exploration results of different configurations in terms of external memory access for DenseNet121 on the four target architectures. The feature of DenseNet is much less external memory access in comparison with other CNNs, therefore we select it as a benchmark to test our reconfiguration methodology. Figure 16 and Figure 17 show that the second target architecture and the third target architecture also have similar configuration effects as in the case of HarDNet. In contrast, Figure 15 and Figure 18 show that HarDNet and DenseNet have much different configuration effects on the first target architecture and the fourth target architecture. Detailed analysis and discussion will be given in the discussion section.

**Figure 19:**
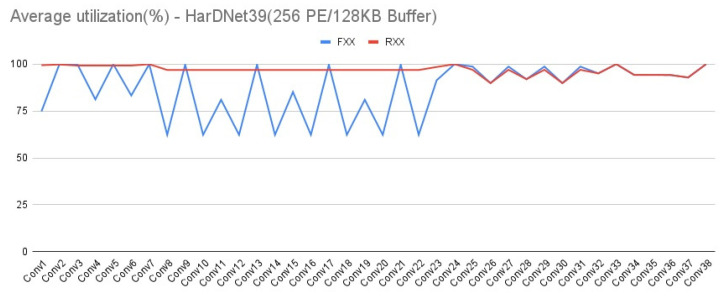
Average PE utilization—HarDNet39 (256 PE/128 KB Buffer).

**Figure 20:**
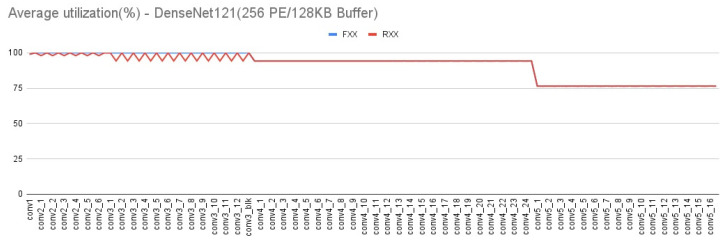
Average PE utilization—DenseNet121 (256 PE/128 KB Buffer).

In addition to the evaluation of external memory access, our platform can also evaluate the PE utilization during the CNN computation. A low utilization rate means more bottleneck of CNN computation, and will damage the CNN performance. Figure 19 and Figure 20 show the utilization rate layer by layer of HarDNet39 and DenseNet121 on the first target architecture, respectively. We can see that in the front layers of HarDNet39 which have a much higher aspect ratio of ifmap dimension, the PE array configuration item can enhance the overall PE utilization effectively. While for DenseNet121, the PE array configuration item is only a little worse on PE utilization in the front layers, but is still quite closed to fully utilization. As for either HarDNet39 or DenseNet121, all the other three target architectures have almost the same PE utilization in comparison with the first target architecture, we do not show these similar results again.

Finally, we synthesis the RTL code of the reconfigurable architecture generated by our platform (Figure 4), and compare our reconfigurable architecture (Figure 6) with the fixed architecture on the area overhead of extra control circuit to show the feasibility of our methodology. In this experiment, each PE includes one 8 bit MAC and one 24 bit accumulation register, the synthesis tool used is Synopsys Design Compiler, and synthesis library used is CBDK_TSMC40_Arm_f2.0 (40 nm). Table 3 shows the comparison result of the four target architecture.

## 5. Discussions

In this section, we analyze and discuss the exploration results in the previous section. For the HarDNet39, we see that either increasing memory capacity or increasing PE array size is effective in reducing external memory access. Compared to the first target architecture, Figure 13 shows that when increasing memory capacity only, all configurations have a significant improvement on reducing DRAM access, although the “RFF” and “RRF” configurations have even worse results than the “FFF” configuration. While when increasing PE array size only, Figure 14 shows that although all configurations still reducing DRAM access, except that the “RFF” and “RRF” configurations have even worse results than the “FFF” configuration, the “FFR”, “RFR”, “FRR” and “RRR” configurations get much worse results than when increasing memory capacity only. Finally, when increasing both memory capacity and PE array size, Figure 12 shows that the “RFF” and “RRF” configurations still have much worse results than the “FFF” configuration, and this target architecture only get a little improvement for all configurations in comparison with the third target architecture that increasing memory capacity only (Figure 13). 

In summary for HarDNet39, among all the configurations, only PE array configuration but without data reuse configuration (“RFF” and “RRF”) get the worst results, integrating both PE array configuration and data reuse configuration (“RFR” and “RRR”) get the best results when increasing memory capacity, and the effect of data reuse configuration (“FFR”, “RFR”, “FRR” and “RRR”) will be much degraded when increasing PE array size only in comparison with when increasing memory capacity only. Among the four target architectures, the third one (increasing memory capacity only) will be the best since it has only a little worse DRAM access in comparison with the fourth target architecture (increasing both memory capacity and PE array size) for all configurations, but it needs only one fourth of the PE array size.

For DenseNet121 which is a neural network with much less external memory access in comparison with other CNNs, only PE array configuration but without data reuse configuration (“RFF” and “RRF”) also get the worst results on all the four target architectures. For the effect of different configurations on DenseNet121, we see that for target architectures with the same memory capacity but different PE array size, Figure 16 and Figure 17 (target architectures 2, 3) show that they have similar configuration effects, and this is the same for Figure 15 and Figure 18 (target architectures 1, 4). While on the effect of memory capacity and PE array size, Figure 18 shows that increasing PE array size only has less DRAM access in comparison with increasing memory capacity only (Figure 17) for all configurations. That is, for a neural network which is featuring on less external memory access, increasing PE array size is a better choice to further reduce DRAM access.

In summary, our platform makes an exploration on the different combinations of configuration issues to investigate their effectiveness, and can be used as a guide to speed up the thorough exploration process on different target architectures.

## 6. Conclusions

In this paper, we propose a reconfigurable architecture and data reuse methodology layer by layer for external memory traffic minimization and PE utilization enhancement of CNNs, and is shown to be effective on the edge device which has limited hardware resources. Especially, the additional control and hardware cost for these configurations is reasonable and executable. The proposed exploration platform can evaluate the effect of different configurations efficiently on different target architectures for different CNNs, and therefore is convenient to evaluate and select approximate CNN and edge devices for the target application.

In the future, making the different configuration items integrate more precisely is the first work to do. In addition, extending the exploration platform for more evaluation items is also ongoing work. After all, implementing the proposed reconfigurable architecture on a promising platform like FPGA will be the most important work to do. Due to limited FPGA storage capability and memory bandwidth as described in research [31], more complex and efficient architecture design, dataflow and data reuse techniques will still be the focus of this future work.

## Figures and Tables

**Table 1:** Target architecture configurations.

	Fixed			Reconfigurable		
	PE Array	Buffer Size	Dataflow, Data Reuse	PE Array	Buffer Size	Dataflow, Data Reuse
**Architecture1**	16 × 16	Input: 64 KB Filter: 64 KB	Output Stationary, Convolutional	256 Total	128 KB Total	Output Stationary, Convolutional + Input + Filter
**Architecture2**	32 × 32	Input:128 KB Filter:128 KB	Output Stationary, Convolutional	1024 Total	256 KB Total	Output Stationary, Convolutional + Input + Filter
**Architecture3**	16 × 16	Input:128 KB Filter:128 KB	Output Stationary, Convolutional	256 Total	256 KB Total	Output Stationary, Convolutional + Input + Filter
**Architecture4**	32 × 32	Input: 64 KB Filter: 64 KB	Output Stationary, Convolutional	1024 Total	128 KB Total	Output Stationary, Convolutional + Input + Filter

**Table 2:** Definition of configurations.

	FFF	RFF	FRF	FFR	RRF	RFR	FRR	RRR
**PE Array**	Fixed	Configure	Fixed	Fixed	Configure	Configure	Fixed	Configure
**Buffer Size**	Fixed	Fixed	Configure	Fixed	Configure	Fixed	Configure	Configure
**Dataflow**	Fixed	Fixed	Fixed	Configure	Fixed	Configure	Configure	Configure

**Table 3:** Overhead of reconfigurable architecture.

	Fixed	Reconfigurable	Overhead
**Architecture1**	39,481.34 μm^2^	40,841.39 μm^2^	3.44%
**Architecture2**	161,263.71 μm^2^	170,336.73 μm^2^	5.63%
**Architecture3**	40,152.52 μm^2^	41,808.79 μm^2^	4.12%
**Architecture4**	157,946.82 μm^2^	165,562.65 μm^2^	4.82%
